# Investigation of stillbirth causes in Suriname: application of the WHO ICD-PM tool to national-level hospital data

**DOI:** 10.1080/16549716.2020.1794105

**Published:** 2020-08-11

**Authors:** Zita D. Prüst, Kim J. C. Verschueren, Gieta A. A. Bhikha-Kori, Lachmi R. Kodan, Kitty W. M. Bloemenkamp, Joyce L. Browne, Marcus J. Rijken

**Affiliations:** a Department of Obstetrics, Division Women and Baby, Birth Centre Wilhelmina’s Children Hospital, University Medical Centre Utrecht, Utrecht University, Utrecht, The Netherlands; b Department of Obstetrics and Gynaecology, Academical Hospital Paramaribo (AZP), Paramaribo, Suriname; c Julius Global Health, The Julius Centre for Health Sciences and Primary Care, University Medical Centre Utrecht, Utrecht University, Utrecht, The Netherlands

**Keywords:** Stillbirths, foetal death, ICD-PM, perinatal mortality, middle-income country, classification

## Abstract

**Background:**

Suriname has one of the highest stillbirth rates in Latin America and the Caribbean. To facilitate data comparison of perinatal deaths, the World Health Organization developed the International Classification of Diseases-10 Perinatal Mortality (ICD-PM).

**Objective:**

We aimed to (1) assess characteristics and risk indicators of women with a stillbirth, (2) determine the timing and causes of stillbirths according to the ICD-PM with critical evaluation of its application and (3) propose recommendations for the reduction of stillbirths in Suriname.

**Methods:**

A hospital-based, nation-wide, cross-sectional study was conducted in all hospitals within Suriname during one-year (2017). The medical files of stillbirths (gestation ≥28 weeks/birth weight ≥1000 grams) were reviewed and classified using ICD-PM. We used descriptive statistics and multiple logistic regression analyses.

**Results:**

The stillbirth rate in Suriname was 14.4/1000 births (n=131 stillbirths, n=9089 total births). Medical files were available for 86% (n=113/131) of stillbirths. Women of African descent had the highest stillbirth rate and two times the odds of stillbirth (OR 2.1, 95%CI 1.4–3.1) compared to women of other ethnicities. One third (33%, n=37/113) of stillbirths occurred after hospital admission. The timing was antepartum in 85% (n=96/113), intrapartum in 11% (n=12/113) and unknown in 4% (n=5/113). Antepartum stillbirths were caused by *hypoxia* in 46% (n=44/96). In 41% (n=39/96) the cause was unspecified. *Maternal medical and surgical conditions* were present in 50% (n=57/113), mostly hypertensive disorders.

**Conclusion:**

Stillbirth reduction strategies in Suriname call for targeting ethnic disparities, improving antenatal services, implementing perinatal death audits and improving diagnostic post-mortem investigations. ICD-PM limited the formulation of recommendations due to many stillbirths of ‘unspecified’ causes. Based on our study findings, we also recommend addressing some challenges with applying the ICD-PM.

**Abbreviations:**

CTG: Cardiotocography; ENAP: Every Newborn Action Plan (ENAP); ICD-PM: The WHO application of ICD-10 to deaths during the perinatal period – perinatal mortality; SBR: Stillbirth rate; SGA: Small for gestational age; WHO: World Health Organization; LMIC: Low- and middle-income countries; FHR: foetal heart rate.

## Background

Stillbirth is one of the most common adverse pregnancy outcomes. It is often related to severe maternal morbidity and associated with long-lasting psychosocial distress for mothers and their families. However, stillbirths often remain hidden from society [1]. The estimated worldwide stillbirth rate (SBR) is 18.4 per 1000 births, yet the numbers vary substantially per country (1.3 to 43.1 per 1000 total births) [2]. Most stillbirths (98%) occur in low- and middle-income countries (LMIC), affecting the most marginalised communities. Therefore, this also makes it an equality and equity issue [1,2]. Although the Sustainable Development Goals do not explicitly state a stillbirth reduction target, it is an essential indicator for the quality of care in pregnancy and childbirth and a sensitive marker of a healthcare system’s strength [3,4]. The World Health Organization’s (WHO) *Every Newborn Action Plan (ENAP)* aims to reduce stillbirths globally, with the target of no more than 12 stillbirths per 1000 total births in every country by 2030 [1]. Despite the endorsement of the ENAP and an increase in the number of studies on stillbirths, most countries have not yet defined a stillbirth reduction target in their national health plans [4,5].

Stillbirths should be systematically assessed to identify risk factors and causes and provide strategies for reducing the SBR [6]. Obtaining reliable statistics is challenging since stillbirths are often poorly documented by the vital registry [4–7]. A literature review [8] revealed that, between 2009 and 2014, more than 81 systems were in place to classify causes of perinatal deaths, complicating cross-country comparison [8]. In response, the WHO developed a universal classification system, the International Classification of Disease 10 Perinatal-Mortality (ICD-PM) to harmonise classifications and facilitate global data comparison on causes of perinatal deaths [9]. A pilot study in South-Africa and the United Kingdom validated the ICD-PM as the global standard for perinatal death classification [10]. Thus far, no countries in Latin America or the Caribbean have applied the ICD-PM to stillbirths.

A previous nationwide study on perinatal outcomes in Suriname, South America, in 2016 and 2017 reported an SBR of 14.8 per 1000. This ranked Suriname with the second-highest SBR of Latin America and the Caribbean [11,12]. The reason for Suriname’s high SBR is unknown. Similar to many other LMIC, no stillbirth registry or classification system is in place, and no perinatal death audits are performed [4,5,11]. To develop an adequate stillbirth reduction strategy, in-depth investigation into stillbirths is necessary to identify risk factors, causes and contributing factors. Therefore, we introduced the WHO ICD-PM tool and applied this to national-level hospital data. This study aimed to (1) assess pregnancy characteristics and risk indicators of women with stillbirths in Suriname, (2) determine the timing and causes of stillbirths according to the ICD-PM and evaluate the applicability of the tool and (3) propose recommendations for the reduction of stillbirths in Suriname.

## Methods

### Study design

A nationwide, hospital-based, cross-sectional study was conducted in all five hospitals in Suriname over 1 year, from 1 January to 31 December 2017.

### Study setting

Suriname is a multi-ethnic, upper-middle-income country on the northeast coast of South America [13]. In 2018, the population counted 575,991 people, of which approximately 90% live in Paramaribo or along the coastline [14,15]. Of the five hospitals in the country, four are located in the capital Paramaribo (including one tertiary hospital), and one in Nickerie, a town on the northwest coast. About 86% of all deliveries in Suriname occur in these five hospitals (one public tertiary facility and four public secondary facilities). The public primary healthcare centres perform 6% of births, mostly in the interior and rural coastal areas. Of the remaining 8%, half are home births and half remain unknown [15]. Suriname has one of the most ethnically diverse populations globally, with each group preserving its own culture [15].

The ethnic distribution in Suriname is Hindustani (27%), Maroon (22%), Creole (16%), Javanese (14%), Mixed (13%), Indigenous (4%), Chinese (1%) and Other (3%) in 2018 [12,14,15]. Suriname’s ethnic diversity reflects its history. Indigenous people, also known as Amerindians, are the original inhabitants of the country. Maroons and Creoles are of (West-) African descent, as they were enslaved and brought to Suriname in the seventeenth and eighteenth centuries. In contrast to Creoles, Maroon people escaped from slavery and fled into Suriname’s interior where they lived separately from the rest of the population for decades. Following the abolition of slavery in 1863, Creoles gained their freedom. They sometimes have mixed African-European (Dutch and British) ancestry. People of Asian descent, Hindustani (from East-India), Javanese (from Indonesia, then a Dutch-ruled colony) and Chinese people, came to Suriname in the late nineteenth century as contract workers. Mixed ethnicities are the result of interchanging identities between almost all ethnicities. Other ethnicities include Brazilians, Caucasians (descendants of Dutch colonists) and a few Lebanese [12,15]. Maroon and Indigenous women belong to the poorest quartile of Suriname [15]. We classified ethnicities with the necessary ethical considerations: the principles of autonomy were embraced (by self-reporting of ethnicity by patients), there were no interventions (observational study), and reporting of disparities aimed to reduce inequity at the beneficence of women with poorer outcomes.

### Eligibility criteria

We included all live births and stillbirths of babies at or beyond 28 weeks of gestation or with a birth weight of ≥1000 grams (WHO definition) in Suriname’s hospitals [9,16]. The SBR was defined as the number of late stillbirths per 1000 total births [9]. Gestational age was determined using early ultrasound examination, as this is a standard procedure during antenatal care visits. If no early ultrasound examination had been performed, later ultrasound examinations or the estimated last menstruation were used to estimate gestational age. Live births during the study period were used as the reference group to analyse risk indicators.

### Variables

Age categories were based on definitions used in previous studies of teenage pregnancies (<20 years) and pregnancies at advanced maternal age (≥35 years) [17,18]. Grand multiparity was defined as four or more previous births beyond 22 weeks of gestation [19]. Ethnicity was self-reported, similar to the national Multiple Indicator Cluster Survey [15]. Moderate anaemia was defined as a haemoglobin level below 100 g/L (6.2 mmol/L) and severe anaemia as a haemoglobin level below 70 g/L (4.3 mmol/L), according to the WHO definition [20]. Preterm birth was defined as a delivery before 37 weeks of gestation [19,21]. Categories of preterm deliveries were set according to the WHO definition: late preterm (32 to 37 weeks) and extremely preterm (below 32 weeks) [21]. A birth weight lower than 2500 grams was considered a low birth weight [19]. Small for gestational age was defined as weight under the 10th percentile according to INTERGROWTH 21^st^ charts [22]. A macerated foetus was defined as a stillbirth with skin and soft tissue changes such as redness, peeling and skin discolouration [9,11]. Congenital malformations were determined by reported macroscopic abnormalities.

### Data collection

In Suriname, midwives and doctors are responsible for the registration of each birth, which is done manually in childbirth books on the maternity wards. Each hospital digitalised the childbirth book of 2017 with the assistance of one of the authors (ZP). The availability of variables was described elsewhere [12]. In brief, basic childbirth data (e.g. maternal age and parity) were available, while information on socio-economic status, BMI, medical history and current pregnancy was unavailable [12].

The medical files were located and examined in detail when it was unclear whether the foetus was born dead or alive. Death certificates were not used for identification of stillbirths, as they are often not completed until several weeks after the birth of a stillborn baby. The medical files of all stillbirths were reviewed and summarised. Maternal and neonatal characteristics and clinical information were entered into Microsoft Office Excel (see supplemental file 1). Early neonatal deaths were not included because the childbirth books did not provide information on neonatal deaths after transfer or discharge of the baby. Two independent clinicians (ZP, GB) classified the stillbirths according to the ICD-PM. If no consensus was achieved, the advice was obtained from an external expert (MR). Autopsy or placental histopathological examinations were not performed for stillbirths, as this is not a standard post-mortem investigation in Suriname.

### Application of the ICD-PM

The ICD-PM classifies perinatal deaths according to a three-step process [9]:

1. Identify the timing of death, which can be either antepartum or intrapartum. Neonatal deaths were not assessed in this study.
2. Assign the causes, with six options (A1-A6) in the antepartum group and seven options (I1-I7) in the intrapartum groups. These ICD-PM groups represent the main causes of foetal deaths and are linked to ICD-10 codes.
3. Identify the main maternal condition affecting the foetus, consisting of five main groups (M1-M5).

Antepartum or intrapartum deaths were distinguished by foetal heart rate (FHR) on admission, cardiotocography (CTG) on the maternity ward, information on cervical dilation and presence of painful uterine contractions. If no information on FHR or stage of labour was available, the timing of death was classified as *‘unable to classify timing’*.

If the cause of the stillbirth could not be determined, it was classified as ‘*unspecified cause’*. Placental abruption was classified as antepartum hypoxia, similar to previous studies [9,10].

Maternal condition *M1* ‘Complications of placenta and membranes’ included placenta praevia, placental abruption and prolapsed cord. The *M4* ‘Maternal medical and surgical conditions’ included maternal conditions such as hypertensive disorders, gestational diabetes and sickle cell disease. When more than one assignable cause was identified (for example, a woman with severe pre-eclampsia, foetal growth restriction and placental abruption), the causes were classified by the first event in the chain: the underlying problem (in this example severe pre-eclampsia) [23]. If there were several, independent maternal factors, the factor contributing most significantly was used for classification.

### Data analysis

Descriptive data analysis consisted of frequency (percentages), mean (standard deviation) and median (interquartile range (IQR)) if variables were not normally distributed. Categorical variables were analysed using cross-tabulations and chi-square test for significance (p < 0.05). Denominator data for assessment of associated risk indicator consisted of all hospital deliveries of live births. We performed no data imputation, as missing data were <5% and assumed to be missed at random.

Univariate binary logistic regression was performed to assess factors associated with stillbirths, reported in odds ratios (OR) with 95% confidence intervals (95% CI). Multiple logistic regression was performed for variables with p < 0.1 in the univariate analysis. We also included variables that were risk indicators reported in previous studies (age, parity and ethnicity [7,12,17,18]). The results of the multiple logistic regression were reported as adjusted odds ratio (aOR). Several possible explanatory variables could not be included in our regression analysis as these variables were not available for the reference population (socio-economic status, residency, BMI or pre-existing maternal conditions and information on current pregnancy). IBM-SPSS version 25 was used for data analysis.

## Results

**Figure 1.**
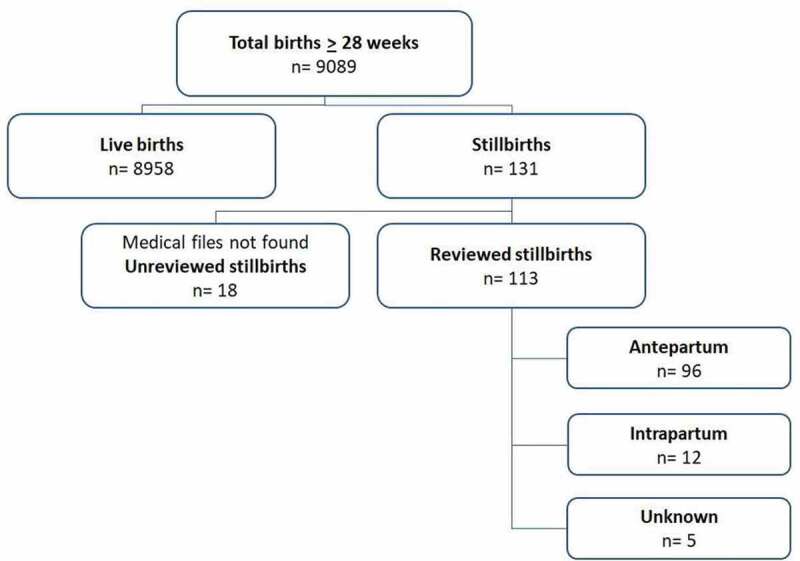
Flowchart of the total births and stillbirths in Suriname in 2017.

In 2017, a total of 9089 babies were born to 8985 women in hospitals in Suriname (Figure 1).

There were 131 stillbirths, resulting in an SBR of 14.4 per 1000 births. One woman with a twin pregnancy delivered two stillborn babies. The total number of deliveries of live births was 8855.

### Characteristics and risk indicators of women with stillbirths

Table 1 displays maternal and foetal characteristics of stillbirths compared to live births. Hospital I, the only referral hospital, had the highest SBR of 26.3 per 1000 births and hospital IV had the lowest SBR of 8.6 per 1000 births. Maternal age (mean 28.4, SD 6.6 years) did not differ between women who experienced a stillbirth and those who did not. The highest SBR was among women of African descent (Maroons and Creole), with 20.2 stillbirths per 1000 total births. Women of Asian descent (Hindustani, Javanese, Chinese) had the lowest SBR with 8.3 stillbirths per 1000 total births.

**Table 1.** Characteristics of stillbirths compared to births in Suriname in 2017.

	Stillbirths n = 131 (%)	Live births n = 8855 (%)	p-value
**Hospital**			
I	54 (41.2)	1998 (22.6)	
II	29 (22.1)	2599 (29.4)	p < 0.001
III	29 (22.1)	2409 (27.2)	
IV	13 (9.9)	1488 (16.8)	
V	6 (4.6)	361 (4.1)	
**Age** (years)			
12–19	15 (11.5)	1254 (14.2)	
20–34	93 (71.0)	6264 (70.9)	p = 0.532
≥35	23 (17.6)	1323 (15.0)	
*Missing*	*0*	*14*	
**Ethnicity**			
African descent	91 (69.5)	4410 (50.7)	
Asian descent	23 (17.6)	2744 (31.5)	
Mixed	13 (9.9)	1153 (13.3)	p = 0.003
Indigenous	4 (3.0)	334 (3.8)	
*Other*	0 (0.0)	60 (0.7)*^a^*	
*Missing*	*0*	*154*	
**Parity**			
0	30 (22.9)	3047 (34.5)	
1–3	69 (52.7)	4625 (52.4)	p < 0.001
≥4	32 (24.4)	1155 (13.1)	
*Missing*	*0*	*28*	
**Antenatal care**			
No	18 (14.9)	*N/A*	
At least one visit	103 (85.1)		-
*Missing*	*10*		
**Insurance**			
Yes	89 (82.4)	*N/A*	-
*No*	19 (17.6)		
*Missing*	*23*		
**Anaemia**			
Severe (Hb <4.3)	6 (6.3)	56 (2.0)	
Moderate (Hb 4.3−6.1)	35 (36.5)	986 (35.1)	p = 0.015
None (Hb ≥6.2)	55 (57.3)	1768 (62.9)	
*Missing*	*35*	*6045*	
**HIV**			
Positive	9 (7.4)	*N/A*	
Negative	112 (92.6)		
*Missing*	*10*		
**Gestational age**			
28–32 weeks	46 (36.8)	151 (1.7)	
32–36 weeks	52 (41.6)	925 (10.5)	p < 0.001
≥37 weeks	27 (21.6)	7758 (87.8)	
*Missing*	*6*	*21*	
**Mode of delivery**			
Spontaneous delivery	123 (93.9)	6577 (74.3)	
Instrumental delivery	1 (0.8)	144 (1.6)	p < 0.001
Caesarean section	7 (5.3)	2134 (24.1)	
*Missing*	*0*	*0*	
**Sex**			
Female	71 (54.6)	4339 (49.0)	
Male	59 (45.4)	4511 (51.0)	p = 0.206
*Missing*	*1*	*5*	
**Birthweight** (grams)			
<1500	52 (40.0)	132 (1.5)	
1500–2500	49 (37.7)	1002 (11.4)	p < 0.001
≥2500	29 (22.3)	7687 (87.1)	
*Missing*	*1*	*34*	

^a^Ethnicity other: Brazilian (n = 44), Caucasian (n = 12), Guyanese (n = 3), Caribbean (n = 1). N/A = not available.

Table 2 presents the multivariable analysis and the factors associated with stillbirth. There was no association between maternal age and stillbirth after adjusting for confounders. Women of African descent had two times the odds of stillbirth compared to women of other ethnicities (aOR 2.1; 95%CI 1.4–3.1), after adjustment for confounders maternal age and parity.

**Table 2.** Multivariate regression analysis for factors associated with stillbirths in Suriname (n = 130).

	Odds ratio (95% CI)	Adjusted odds ratio
**Age**		*Adjusted for parity and ethnicity*
<20 years (vs. 20–34 years)	0.81 (0.47–1.40)	1.00 (0.56–1.84)
≥35 years (vs. 20–34 years)	1.17 (0.74–1.86)	0.90 (0.55–1.47)
**Parity**		*Adjusted for maternal age and ethnicity*
Primiparous (vs. para 1–3)	0.66 (0.43–1.01)	0.72 (0.45–1.13)
Para ≥4 (vs. para 1–3)	1.86 (1.22–2.84)	1.48 (0.92–2.37)
**Ethnicity**		*Adjusted for maternal age and parity*
African descent (vs. all other ethnicities)	2.29 (1.58–3.33)	2.11 (1.43–3.11)
Asian descent (vs. all other ethnicities)	0.46 (0.29–0.73)	0.52 (0.32–0.81)
**Anaemia** (Hb ≤6.1 mmol/L)		*Adjusted for maternal age, parity and ethnicity*
Yes (vs. no anaemia)	1.27 (0.84–1.91)	0.95 (0.62–1.47)

Medical files were available in 86.3% of stillbirths (n = 113/131) (Figure 1). The timing and causes of death of the remaining 18 stillbirths could not be determined. Stillbirths were small for gestational age (SGA) in 26.5% (n = 30/113) of cases. However, foetal weight percentile could not be determined in 34.5% (n = 39) of cases due to unreliable pregnancy dating or timing of death. Maceration was described in 48.7% (n = 55) of stillbirths and congenital abnormalities in 8.0% (n = 9) of stillbirths (Table 1). The stillbirth occurred at home or during transportation to the hospital in 67.3% (n = 76/113) of cases and after hospital admission in 32.7% (n = 37/113) of cases. In total, there were seven women with a stillbirth who delivered by caesarean section. In two cases the stillbirth was not yet diagnosed. In five cases the foetal death was known, and caesarean section was performed on a maternal indication, of which there was one perimortem caesarean section for a woman who died due to a cardiac arrest.

### Classification of stillbirths

**Figure 2.**
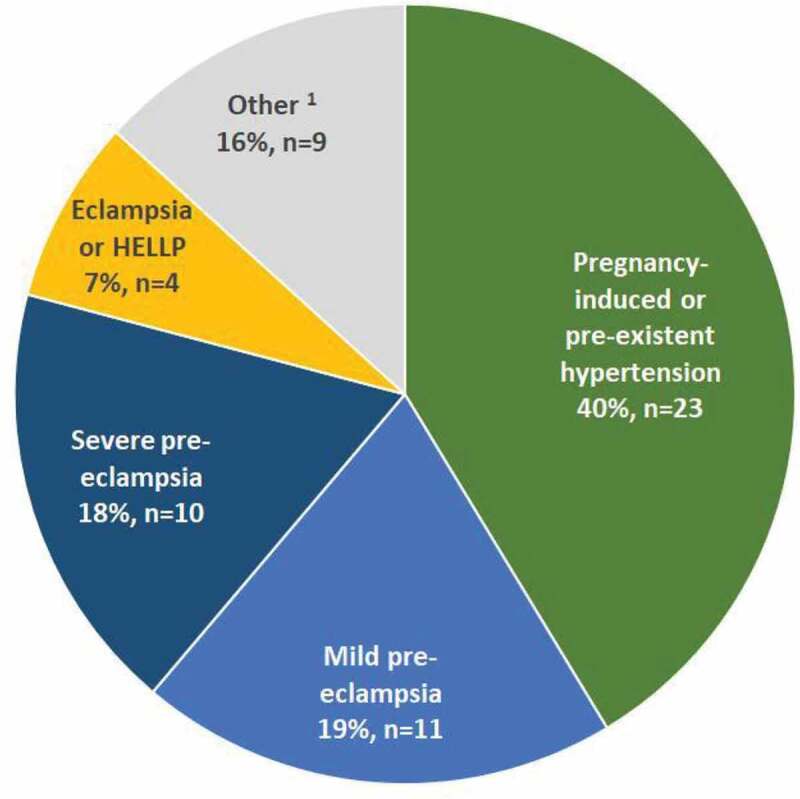
Women with stillbirths with a maternal condition classified as M4 (Maternal medical and surgical conditions), n = 57 (includes four women with unknown timing of death).

Of the 113 stillbirths that were classified, 85.0% (n = 96) occurred antepartum and 10.6% (n = 12) intrapartum. In 4.4% (n = 5) of cases, the timing of death could not be determined despite the availability of the medical file (Figure 1). The classification of stillbirths, according to the ICD-PM, is reported in Table 3. In 34.5% (n = 39/113) of all classified stillbirths, the cause remained unknown. Women had hypertensive disorders of pregnancy in 42.5% (n = 48) of cases (Figure 2) and 23.0% (n = 26) of stillbirths were due to a placental abruption.

**Table 3.** Classification of stillbirths in Suriname, according to the ICD-PM.

	Maternal medical condition					
	M1 Complications of placenta, cord and membranes	M2 Maternal complications of pregnancy	M3 Other complications of labor and delivery	M4 Maternal medical and surgical conditions	M5 No maternal condition	Causes Total (%)
Causes of antepartum deaths						
A 1: Congenital malformations, deformations and chromosomal abnormalities	1				1	**2(2.1)**
A 2: Infection				1		**1(1.0)**
A 3: Antepartum hypoxia	11	1	1	31		**44(45.8)**
A 4: Other specified antepartum disorder		1		1		**2(2.1)**
A 5: Disorders related to foetal growth	1			2	5	**8(8.3)**
A 6: Foetal death of unspecified cause		1		13	25	**39(40.6)**
Causes of intrapartum deaths						
I 1: Congenital malformations, deformations and chromosomal abnormalities				1		**1(8.3)**
I 2: Birth trauma						-
I 3: Acute intrapartum event	2	1	3	4	1	**11 (91.7)**
I 4: Infection						-
I 5: Other specified intrapartum disorder						-
I 6: Disorders related to foetal growth						-
I 7: Intrapartum death of unspecified cause						-
Unknown timing of death						
Total				**4**	**1**	
Maternal condition total (%)	**15** **(13.3)**	**4** **(3.5)**	**4** **(3.5)**	**57** **(50.4)**	**33** **(29.2)**	**113** **(100)**

Includes antepartum and intrapartum stillbirths, as well as stillbirths with an unknown timing of death (n = 4/5 unknown timing with M4).^1^ Other consists of women with (gestational) diabetes (n = 3), sickle cell disease (n = 3), HIV (n = 1), Zika virus (n = 1), car-accident (n = 1).

#### Antepartum (n = 96)

The leading cause of antepartum stillbirths was ‘Antepartum hypoxia (A3)’ (45.8%, n = 44), which was most frequently associated with ‘Maternal medical and surgical conditions (M4)’ (70.5%, n = 31) and *‘*Complications of placenta, cord and membranes (M1)’ (25.0%, n = 11). The second main group consisted of stillbirths of ‘Unspecified cause (A6)’ (40.6% of all antepartum stillbirths, n = 39/96), mostly to women without a medical condition (64.1%, n = 25/39) (Table 3).

#### Intrapartum (n = 12)

The majority of deaths during the intrapartum period were caused by an ‘Acute intrapartum event (I3)’ (91.7%, n = 11) (Table 3). In four cases, there was a placental abruption during labour and in three cases, obstructed labour, e.g. complicated breech delivery.

#### Maternal condition (n = 80)

The majority of stillbirths (70.8%, n = 80) were classified with at least one maternal condition. The most frequently determined maternal conditions were ‘Maternal medical and surgical conditions (M4)*’* (71%, n = 57/80)

### Classification difficulties

**Figure 3.**
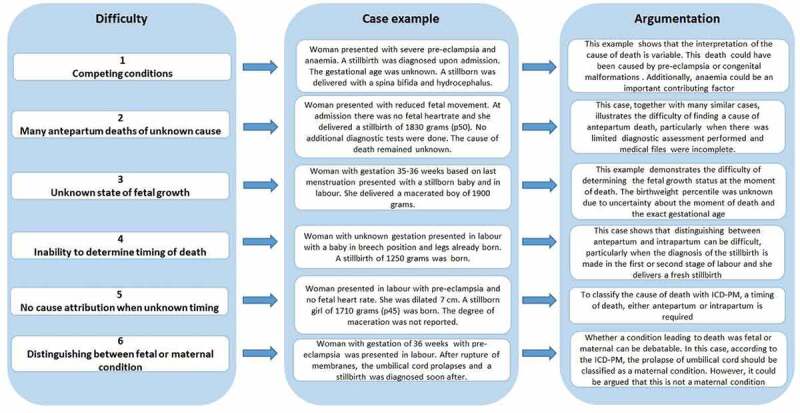
Challenges encountered in the application of the ICD-PM in Suriname.

Figure 3 illustrates the challenges encountered in this study during ICD-PM classification. The most critical difficulties were as follows: (1) the fact that one attributable cause of death had to be assigned, while there were often competing conditions within the chain-of-events (e.g. hypertensive disorders, growth restriction and placental abruption); (2) the large proportion of antenatal deaths of unknown cause; (3) the difficulty of determining foetal growth (when gestational age or timing of death was unknown); (4) the inability to reliably determine the timing of death (antepartum or intrapartum); (5) the inability to assign a cause of death to stillbirths of unknown timing and (6) discussion of whether certain conditions should be classified as a maternal condition when they are a foetal condition (umbilical cord prolapse).

## Discussion

### Main findings

This study is the first to apply the WHO ICD-PM tool on stillbirths in the Americas. Stillbirths have not previously been studied in Suriname, and the SBR of 14.4 per 1000 births found in this study is higher than in most other LMIC in Latin America and the Caribbean [11]. Women of African descent were at higher risk of a stillbirth compared to women of all other ethnicities. Stillbirths occurred predominantly during the antepartum period (85%) and before arrival at the hospital (67%). When a stillbirth cause was determined, the death was mostly attributable to hypertensive disorders. However, a major group of antepartum stillbirths remained of unknown cause (39%), often due to a lack of information and poor diagnostic evaluation post-mortem.

### Interpretation

The SBR among women of African descent in our study is two and a half times higher than among women of Asian descent. Previous studies in Suriname have also reported substantial ethnic disparities in maternal deaths and stillbirths, with similar increased odds of adverse outcomes among women of African descent [12]. These differences may reflect inequity within the healthcare system and need further investigation [12,24]. Body Mass Index (BMI) and socio-economic circumstances could have also contributed to the ethnic disparity seen in the SBR. Adjustment for these variables (BMI, level of income, education, place of residence) was not possible as they were not available from routinely collected data. Well-designed prospective studies are urgently needed to identify high-risk women and develop effective stillbirth prevention strategies.

A gradually shifting pattern is seen globally, from high SBRs with mostly intrapartum deaths in low-income countries to low SBRs with mostly antepartum deaths in high-income countries [10,25–29] (see appendix file 2). The SBR and timing of stillbirth can be incorporated into the ‘obstetric transition’ framework, which describes five stages in which countries shift from high maternal mortality and fertility and many communicable diseases (stage I–II) to low maternal mortality and fertility and more non-communicable diseases [30]. These stages help to understand the context and provide justification for appropriate interventions for reducing maternal (and perinatal) mortality [30]. In various low-resource settings, half of stillbirths (51%) occurred in the intrapartum period. In addition to ensuring access to care, these findings emphasise the need to improve the quality of intrapartum care (e.g. foetal monitoring) to reduce perinatal mortality [25–27].

In middle-income countries, the proportion of antepartum (80%) and intrapartum (20%) stillbirths suggests that intrapartum quality of care is gradually improving and antenatal quality of care improvement is essential for further reduction of perinatal mortality [10,28,29]. Suriname follows these trends with 85% of stillbirths in the antepartum period and serves as an example of a country, which has largely overcome barriers for women to access care. However, a high maternal and perinatal mortality remains due to suboptimal quality of care [15,24]. In the United Kingdom, a high-income country, the SBR is low, and the majority of stillbirths occur in the antepartum period (91%) [10]. Advanced maternal age, rare (pre-existent) and non-communicable maternal diseases and congenital malformations are the main contributors to stillbirths in this high-income setting.

Previous studies have reported a wide variation in the distribution of causes, even between countries with similar settings and economies [10,25–29]. While we classified placental abruption as antepartum hypoxia, similar to the ICD-PM pilot study in the United Kingdom and South Africa (2016) [10], a later study in South Africa (2018) classified placental abruption as *‘Other specified antepartum disorders (A4)’* [28]. Consequently, this led to *‘Antepartum hypoxia (A3)’* being classified as the stillbirth cause in South Africa in 53% in the first study (2016) compared to 0% in the second study (2018) [10,28]. Variations in cause identification and diverse interpretations of the ICD-PM classification make stillbirth study comparisons within and between settings difficult.

A large proportion of stillbirths in Suriname (39%) remained unknown due to an unknown cause or unknown timing after application of the ICD-PM tool, despite the availability of medical files and adequate documentation of the chain of events once admitted to the hospital. Previous studies on applications of the ICD-PM have reported similar figures with *unknown causes* representing between 89% of antepartum stillbirths in low-income settings [26] and 38% in high-income settings [10] (see supplemental file 2). A large amount of *unknown causes* hinders healthcare providers, researchers and policymakers, in establishing interventions to reduce the number of stillbirths. Explanations for the high proportion of *unknown causes* in Suriname were (1) the lack of diagnostic assessment tools (no autopsy, no histological examination of the placenta, no swabs or cultures and minimal maternal blood tests) used in stillbirths, and (2) the lack of stillbirth audits. Determination of causes remains challenging due to the pathophysiological interaction between mother, foetus and placenta and multiple co-existing conditions that can contribute [31,32]. Previous studies have shown that diagnostic assessments, such as post-mortem autopsy or minimal invasive perinatal autopsy, reduced the percentage of unexplained deaths [32–35]. Global consensus on standardisation of necessary diagnostic assessments in stillbirths would not only improve classifications globally but also motivate healthcare providers, researchers and policymakers to reduce preventable stillbirths. It is vital to consider, not only the direct cause leading to death but the chain of events as well. This includes preliminary medical conditions and contributing substandard care factors. The ICD-PM needs to be extended with the addition of perinatal audit to develop country-specific strategies and recommendations for stillbirth reduction.

Maternal conditions occurred in 71% of stillbirths in Suriname, in 59% of stillbirth in South Africa and in 76% of stillbirths in low-resource settings [26,28]. A systematic review on global causes and contributing factors of stillbirths in 2018 [36] reported that maternal conditions contribute to only 37% of global stillbirths, which is much lower than in the studies applying the ICD-PM classification [25–29,36]. Mirroring a recently published commentary by Lavin et al. [31], these different numbers in the attribution of maternal conditions reflect the variance between classification systems on what conditions are classified as a ‘maternal condition’. For example, the ICD-PM maternal condition group *M1 Complications of placenta, cord and membranes* includes cases of ‘cord around neck’ and ‘prolapsed cord’. However, arguably this is not a maternal condition. It is crucial to enable accurate reporting of maternal complications in stillbirths to target and invest in the right actions [31].

### Challenges using ICD-PM

Based on our study findings, we make the following recommendations to address the current challenges with applying the ICD-PM (Figure 3):

1. To make recommendations on wherein the chain of events the cause of stillbirth classification should be set, especially when there are multiple contributing factors (36).
2. To add a checklist of the minimal data (events and outcomes (not mutually exclusive)) that are required to use the ICD-PM classification.
3. To elaborate on the definition of antepartum versus intrapartum stillbirth and make recommendations on the classification of causes when the timing of death is unclear.
4. To gain a global consensus on which conditions/events should be included as a maternal complication.

Addressing these issues will ensure better quality and more consistent reporting, necessary to signiﬁcantly impact the current global priority of reducing preventable perinatal deaths.

### Limitations

This study’s main limitation was that underreporting might have occurred. This study collected data from all national hospitals, that cover 86% of all deliveries in the country. Therefore, results could be an under- or overestimation of the SBR in Suriname (depending on the SBRs in primary healthcare facilities and at home). The documentation in the childbirth books was generally complete; however, stillbirths could have been missed (e.g. an infant could have been recorded as a neonatal death in case a 1-minute APGAR score of 1 was reported while the baby was stillborn). Additionally, this study did not include early neonatal deaths, although these are generally closely related to pregnancy and delivery and should be investigated to assess the whole burden of perinatal mortality.

Furthermore, only basic information was available from the reference group of live births. Since no perinatal registry is yet in place, no information was available on demographics, general or obstetric history, or current pregnancy. This lack of data hindered analysis of risk factors for stillbirths. It is crucial to establish a high-quality national data registry, which includes the abovementioned variables for future studies on risk factors of severe maternal and perinatal outcomes. We believe that our study can serve as an example for other LMIC. We emphasise the importance of (1) establishing a perinatal data registry with a list of essential variables, and (2) discussing and addressing stillbirth classification challenges in order to obtain reliable and valuable statistics for the reduction of perinatal mortality.

Even though this study provided classification and indications regarding causes of death, our experience with ICD-PM was that it was insufficient for generating the evidence needed to inform researchers, healthcare providers and policymakers as to why these stillbirths occurred and how the burden of stillbirths can be reduced in Suriname.

To develop more country-specific strategies for the reduction of stillbirths, more insight into the causes of stillbirth is necessary. Recommendations include (1) accurate, prospective monitoring of stillbirths (2) installation of a perinatal audit committee and (3) development of national guidelines for diagnostic assessment of stillbirth, including routine blood tests, cultures, radiology and minimally invasive autopsy.

## Conclusion

Suriname has a high stillbirth rate of 14.4 per 1000 births and requires a stillbirth reduction action plan. Women of African descent are at the highest risk of stillbirth, and further studies need to assess socio-economic background and health services related factors contributing to the high SBR and ethnic disparities. The majority of stillbirths occurred before hospital admission, and hypertensive disorders formed the primary cause of stillbirths, emphasising the importance of improving the quality of antenatal care services. A significant proportion of stillbirths were classified as ‘unspecified’, limiting further development of recommendations for the reduction of stillbirths in Suriname. Perinatal death audits, post-mortem investigations and guidelines for the diagnostic assessment are necessary to improve the knowledge about the causes and development of recommendations. Addressing the challenges described in this study may improve ICD-PM feasibility and applicability in Suriname and other LMIC settings.

## Supplementary Material

Supplemental Material

Supplemental Material

## Paper context

The middle-income country, Suriname, is a South-American country with one of the highest stillbirth rates of the region. This is the first study to assess late stillbirth causes according to the WHO ICD-PM in the Americas. This article provides risk-indicators for stillbirths in Suriname, the timing and causes of stillbirths, and illustrates the challenges of the ICD-PM classification. To improve identification of causes and reduce stillbirths, perinatal audits and post-mortem guidelines for investigation are needed.
