# Supplementary material for: Investigation of stillbirth causes in Suriname: application of the WHO ICD-PM tool to national-level hospital data
Source: Glob Health Action. 2020 Aug 11;13(1):1794105. doi: 10.1080/16549716.2020.1794105 (PMC7480654; doi:10.1080/16549716.2020.1794105)
Supplement: Supplemental Material [file ZGHA_A_1794105_SM2846.docx]

| **Variables** | **Selection options** |
| --- | --- |
| **Case summary** |  |
| **Maternal age (years)** |  |
| **Ethnicity** | African descendant/Hindustani/Javanese/Mix/Indigenous/Chinese/Other |
| **Insurance** | Yes / No |
| **Gravida** |  |
| **Parity** |  |
| **Abortions** |  |
| **Type of Pregnancy** | Singleton/Twins/Triplets |
| **Antenatal care** | Yes/no |
| **Antenatal care visits** |  |
| **Gestational age at first antenatal care visit** |  |
| **Maternal HIV status** | Positive/Negative/Not tested |
| **HIV Medication if positive** |  |
| **Hepatitis B (HbsAg)** | Positive/Negative/Not tested |
| **VRDL** | Positive/Negative/Not tested |
| **Date of birth baby** |  |
| **Time of Delivery (hh:mm)** |  |
| **Gestational age (weeks)** |  |
| **Gestational age (days)** |  |
| **Sex** | Female/Male |
| **Birthweight (grams)** |  |
| **Method of gestational age assessment** | Early ultrasound/Late ultrasound/last menstruation/fundal height |
| **Gestational age at moment of death** |  |
| **Birth weight percentile at moment of death** |  |
| **Place of delivery** |  |
| **Mode of delivery** | Spontaneous delivery/Instrumental delivery/Caesarean Section |
| **Indication of SC** |  |
| **Induction of Birth** | Oxytocin/misoprostol/Foley catheter |
| **Highest diastolic blood pressure** |  |
| **Hypertensive disorder** | PIH/moderate pre-eclampsia/severe pre-eclampsia/eclampsia |
| **Antenatal hemoglobin (mmol/L)** |  |
| **Sickle cell** | No/yes/not tested, if yes, add type of sickle cell |
| **Pre- or inter-hospital death** | Pre-hospital/inter-hospital/unable to determine |
| **Cervical dilatation on admission (cm)** |  |
| **Spalding** |  |
| **Macerations** | Fresh/Macerated (and degree)/not stated |
| **Timing of death** | Antepartum/Intrapartum/unable to classify |
| **ICD-PM groups for perinatal death** | Antepartum: A1/A2/A3/A4/A5/A6  Intrapartum: I1/I2/I3/I4/I5/I6/I7 |
| **ICD-10 broad category** | Matching ICD-10 code |
| **Other disease or conditions in fetus** |  |
| **Comments** |  |
| **ICD-PM group for Maternal condition** | M1/M2/M3/M4/M5 |
| **Other disease or condition mother** | Matching ICD-10 code |
| **Pre-existing disease** |  |
| **If yes, medication** |  |
| **Comments** |  |
|  |  |

**Supplementary file 1.** Microsoft excel variables used in Suriname for data extraction and classification of stillbirths according ICD-PM
