# Supplementary material for: Investigation of stillbirth causes in Suriname: application of the WHO ICD-PM tool to national-level hospital data
Source: Glob Health Action. 2020 Aug 11;13(1):1794105. doi: 10.1080/16549716.2020.1794105 (PMC7480654; doi:10.1080/16549716.2020.1794105)
Supplement: Supplemental Material [file ZGHA_A_1794105_SM2845.docx]

**Supplementary file 2.** Literature overview of all studies conducted on perinatal mortality using the ICD-PM

|  | | | **High-income** | **Middle-income** | | | | **Low-income** | | |
| --- | --- | --- | --- | --- | --- | --- | --- | --- | --- | --- |
|  | | | **United Kingdom**  **(2016)** | **South-Africa (2016)** | **South Africa (2018)** | **Sri-Lanka**  **(2017)** | **Suriname (2020)** | **Multi-country^2^**  **(2019)** | **Zambia**  **(2019)** | **Tanzania (2020)** |
| **Total perinatal deaths, n=** | | | 9067 | 689 | 26810 | 291 | N/A | N/A | 75 | 661 |
| Total stillbirths, n= | | | 4834 | 418 | 19344 | 205 | 131 | 1267 | 32 | 413 |
| Total neonatal deaths, n= | | | 4233 | 271 | 7466 | 86 | N/A | N/A | 43 | 248 |
| **Perinatal death rate per 1000 births** | | | N/A | N/A | N/A | N/A | N/A | N/A | N/A | 71 |
| **Stillbirth rate per 1000 births** | | | N/A | N/A | N/A | N/A | 14 | Malawi: 20.3  Zimbabwe 34.7  Kenya 38.8 | N/A | 44 |
| **Timing** | **Antepartum** | | 91% | 82% | 81% | 97% | 85% | 42% | 9% | 31% |
|  | **Intrapartum** | | 8% | 18% | 19% | 3% | 11% | 51% | 33% | 31% |
|  | **Unknown** | | 0% | 0% | 0% | - | 4% | 7.3% | 57% | 38% |
| **Main causes**  **Antepartum deaths** | | **A1**  **A2**  **A3**  **A4**  **A5**  **A6** | 22%  ?  ?  ?  15%  38% | ?  ?  53%  ?  ?  42% | 3%  3%  0%  19%  8%  68% | 14%  4%  42%  18%  5%  17% | 2%  1%  46%  2%  8%  39% | 2%  9%  0%  0%  0%  89% | 14%  0%  14%  0%  0%  71% | 3%  1%  46%  0%  0%  50% |
| **Main causes**  **Intrapartum deaths** | | **I1**  **I2**  **I3**  **I4**  **I5**  **I6**  **I7** | ?  ?  65%  ?  ?  ?  ? | ?  ?  93%  ?  ?  ?  ? | 5%  0%  69%  1%  13%  2%  10% | 72%  0%  14%  0%  0%  14%  0% | 8%  0%  92%  0%  0%  0% | 4%  0%  31%  4%  0%  0%  61% | 16%  0%  84%  0%  0%  0%  0% | 2%  0%  67%  0%  0%  1%  30% |
| **Main**  **Maternal condition** | | **M1**  **M2**  **M3**  **M4**  **M5** | 21%  11%  ?  ?  50% | ?  ?  18%  26%  36% | 18%  5%  21%  33%  31% | ?  ?  ?  35%  ? | 13%  4%  4%  47%  28% | 27%  10%  26%  14%  24% | 1%  9%  54%  4%  35% | 12%  6%  9%  18%  34% |
| **Legend**  ^1^ Twenty-nine countries, HIC and LMIC ^2^ Kenya, Malawi, Sierra Leone and Zimbabwe  Abbreviations**:** A1= Congenital malformations, deformations and chromosomal abnormalities, A2= infection, A3= Antepartum hypoxia, A4= Other specified antepartum disorder, A5= Disorders related to fetal growth, A6= unspecified cause. I1= Congenital malformations, deformations and chromosomal abnormalities, I2 = birth trauma I3= Acute intrapartum event, I4= Infection, I5= Other specified intrapartum disorders, I6= Disorders related to fetal growth, I7= Unspecified cause. M1= Complications of placenta, cord and membranes**,** M2= Maternal complications of pregnancy**,** M3=Other complications of labor and delivery, M4= Maternal medical and surgical conditions, M5= No maternal condition identified | | | | | | | | | | |
